# A fatal case of *Nocardia otitidiscaviarum *pulmonary infection and brain abscess: taxonomic characterization by molecular techniques

**DOI:** 10.1186/1476-0711-8-11

**Published:** 2009-04-14

**Authors:** Ana Isabel Pelaez, Maria del Mar Garcia-Suarez, Angel Manteca, Ovidio Melon, Carlos Aranaz, Rafael Cimadevilla, Francisco Javier Mendez, Fernando Vazquez

**Affiliations:** 1 Universidad de Oviedo. Area de Microbiologia, Departamento de Biologia Funcional, Facultad de Medicina, Oviedo, Spain; 2 Department of Biochemistry and Molecular Biology, University of Southern Denmark, Campusvej 55, Odense M, DK-5230, Odense, Denmark; 3 Servicio de Geriatria-Medicina Interna, Hospital Monte Naranco, Oviedo, Spain; 4 Servicio de Microbiologia, Hospital Central de Asturias, Oviedo, Spain; 5 Servicio de Microbiologia, Hospital Monte Naranco, Oviedo, Spain

## Abstract

We report on a rare case of pulmonary Nocardiosis and brain abscess caused by *Nocardia otitidiscaviarum *in an elderly woman with chronic obstructive pulmonary disease. Taxonomic identification involved phenotypic testing, restriction fragment length polymorphism (RFLP), and complete 16S rRNA gene sequencing.

## Case report

An 85-year-old female living in a rural area with a history of hypertension, coronary disease, and severe to very severe chronic obstructive pulmonary disease (COPD) was admitted to hospital with increasing dyspnea, cough, whitish sputum, and pleuritic chest pain on her right side. She had been on treatment with prednisone for eighteen months, after which treatment was switched to deflazacort (15 mg/day) two months prior to suffer two COPD exacerbations. At admission, the patient presented a Barthel Index of 60; her temperature was 37.3°C with 89% oxygen saturation, while breathing ambient air. Spirometric examination demonstrated severe airway obstruction with a forced vital capacity (FVC) of 1.2 L, forced expiratory volume in 1 s (FEV1) of 0.53 L, and FEV1/FVC of 44%. She had diminished breathing sounds and inspiratory crackles in the lower half of both lungs. Physical and neurological examinations were unremarkable.

**Figure 1:**
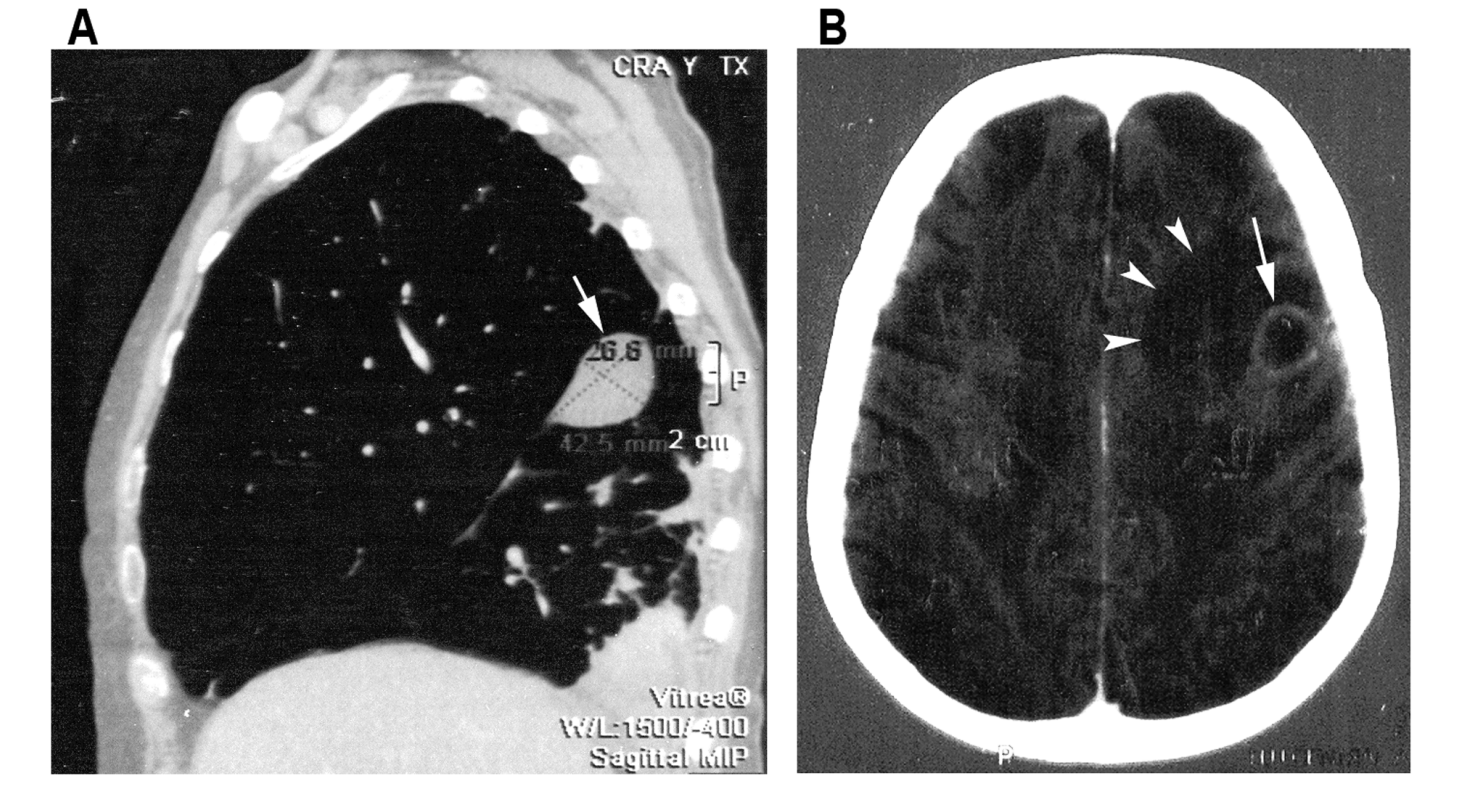
**(A) Computed tomography of the chest showing multiple pulmonary nodules, a small collection of fluid in the major fissure (arrow) and a small pleural effusion on the right**. **(B) **Computed tomography of the brain showing a nodular lesion in the left frontoparietal lobe (arrow) with perilesional vasogenic edema (arrowheads).

Laboratory analyses yielded the following: hemoglobin concentration, 10.7 g; WBC, 22800 (92% neutrophils, 2% lymphocytes); platelet count, 518000; creatinine concentration, 0.92 mg/dl; glycemia, 180 mg/dl, and urinary protein, 80 mg/dl. Chest computed tomography (CT) revealed an interstitial distribution of multiple pulmonary nodules, fluid in the major fissure, and a small pleural effusion (Figure 1A). A contrast-enhanced CT scan of the head revealed three nodular lesions in the left frontoparietal lobe with perilesional vasogenic edema, highly suggestive of brain abscess (Figure 1B).

Repeated sputum and blood cultures were negative. Pleural fluid was obtained by puncture and microscopic examination revealed numerous polymorphonuclear leucocytes and modified acid fast positive, filamentous-branched, gram-positive rods. The growth from the specimen, which had been cultured on blood and chocolate agar plates displayed dry, irregular, adherent, white colonies after 72 h incubation at 37°C. The isolate was initially characterized on the basis of routine phenotypic test results such as colonial morphology, positive Gram stain; positive catalase test; growth in nutrient broth with lysozyme; reduction of nitrate; hydrolysis of xanthine, hypoxanthine and urea, and the inability to hydrolyze casein and tyrosine [1]. The enzymatic profile obtained by API ZYM (BioMerieux, Marcy l'Etoile, France) exhibited negative reactions to valine arylamidase, N-acetyl-β-glucosaminidase, and α-mannosidase. Antimicrobial susceptibility testing was performed simultaneously by Kirby Bauer disc diffusion [2] and E-tests (AB Biodisk, Sweden), following the protocol described by Martin *et al*., 2004, for other actinomycetes (*Streptomyces*) [3]. A suspension of the microorganism, with turbidity equivalent to 1.0 McFarland standard, was inoculated (150 μl/plate) by confluent swabbing on Mueller-Hinton agar (MHA) plates. A maximum of two E-test strips were applied to each MHA plate, which were then incubated at 35°C for 3 days. MICs were determined according to manufacturer's guidelines. Susceptibility results were as follows: penicillin G > 256 μg/ml; ampicillin > 256 μg/ml; imipenem 0.75 μg/ml; vancomycin 32 μg/ml; erythromycin 64 μg/ml; linezolid 0.5 μg/ml; trimethoprim/sulphamethoxazole (TMP/SMX) 0.75 μg/ml; gentamycin 0.25 μg/ml; amikacin 0.75 μg/ml, and ciprofloxacin 0.50 μg/ml.

After a tentative diagnosis of pulmonary nocardiosis, the patient was treated with TMP/SMX (2400/480 mg/day) and imipenem (4 g/day iv) for ten days. However, due to tonic-clonic convulsions, the imipenem was switched to oral linezolid 600 mg/12 h and dexamethasone 10 mg-bolus followed by 4 mg iv every 6 h. The patient developed anemia and thrombocytopenia after one week, and linezolid was therefore discontinued. The patient expired one month after admission. At autopsy, specimens from pleural fluid and the cerebral abscess were taken and cultured. *Nocardia *growth was observed and confirmed by subculture and microscopy: colonies had a brown velvety appearance, branching filaments were visible microscopically (0.5–1.2 μm diameter), that often fragment into rod- to coccoid shaped forms.

In order to characterize the *Nocardia *isolates from pleural fluid and the brain abscess, the 16S rRNA gene (1500 bp) was amplified. The 16S rRNA gene from both isolates was amplified using the primers pA: 5'-AGAGTTTGATCCTGGCTCAG-3' and pH: 5'-AAGGAGGTGATCCAGCCGCA-3'. PCR was performed using a reaction mixture containing 1× PCR buffer; 0.2 mM (each) dATP, dTTP, dGTP, and dCTP; 0.2 mM of each primer; 2 U of EcoTaq DNA polymerase (Ecogen, TaKaRa Biotechnology, Japan), and ultrapure water to a final volume of 50 μl. PCR amplification was performed in a DNA thermal cycler (MJ Research Inc., MA, USA) and included initial denaturation at 94°C for 5 min, followed by 30 cycles of amplification (94°C for 1 min, 57°C for 1 min, and 72°C for 2 min) followed by a 10-min extension at 72°C. PCR products were visualized by 0.8% agarose gel electrophoresis and ethidium bromide staining. Bands corresponding to 16S rRNA gene were purified using the GFX™ PCR DNA and Gel Band Purification Kit (Amersham Biosciences, NY, USA) following the manufacturer's instructions. In order to obtain preliminary information about both isolates the 16S rRNAs were analyzed by restriction fragment length polymorphism (RFLP) with *Hae*III and *Alu*I restriction endonucleases (TaKaRa Biotechnology) [4]. An aliquot of each of the PCR-amplified 16S rRNA genes was digested at 37°C for 4 h and the resulting restriction fragments were separated on a 3% agarose electrophoresis gel. The restriction patterns obtained after digestion were identical in both strains (data not shown), strongly suggesting that the two isolates corresponded to the same microorganism. After that, and with the aim of confirming or refuting, the identity between both isolates, the full 16S rRNA amplified genes were sequenced. Sequencing was performed using the pA and pH primers (see above) and the BigDye^® ^Terminator v3.1 Cycle Sequencing Ready Reaction Kit using an ABI Prism™ 3100 Genetic Analyzer (PerkinElmer Inc., NY, USA) to electrophorese the sequence product. The Sequence Scanner v1.0 (Applied Biosystems Foster City, CA, USA) and BioEdit (v5.0.6) programs were used to analyze the sequence data thereby derived. Sequencing data were queried against sequences in the GenBank  and EMBL  databases using the BLASTN program. The partial sequences of the 16S rRNA genes from these isolates (1,450 bp; GenBank accession no. EU203569 and EU203570) shared maximum identity (100%) with the 16S rRNA from *Nocardia otitidiscaviarum *strain DSM 44565 (GenBank accession no. AF430068), 99.9% identity with *N. otitidiscaviarum *type strain DSM 43242^T ^(GenBank accession no. AF430067) and 98% with other *Nocardia *species such as *Nocardia nova *(GenBank accession no. AB162789). An alignment of the 16S rRNA sequences from both isolates with DSM 43242^T ^type strain displayed a 99.9% identity (only one base difference). This allowed the identification of the pleural fluid and the cerebral abscess isolates as *N. otitidiscaviarum*.

**Figure 2:**
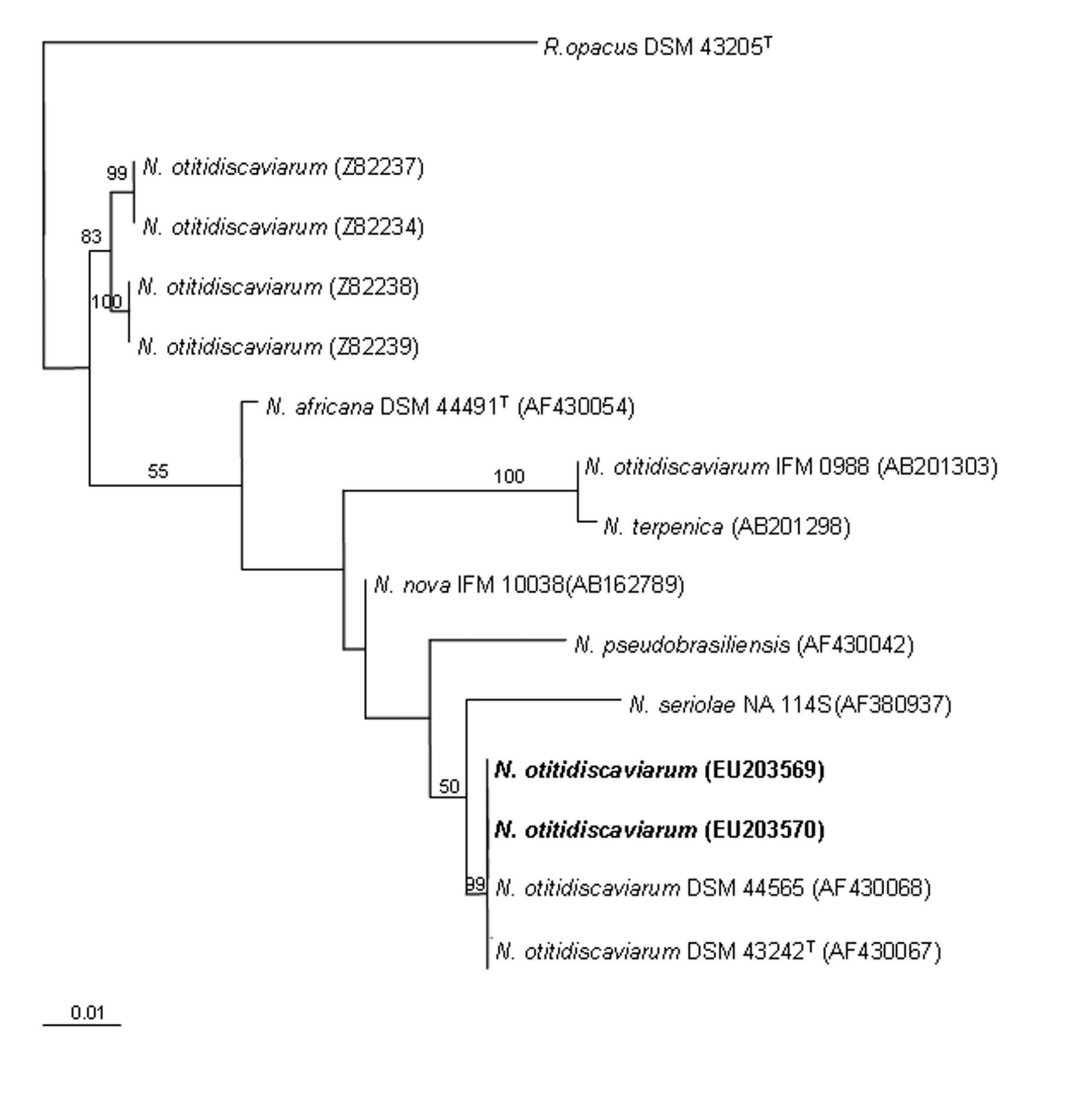
**Phylogenetic tree based on 16S rRNA gene sequences of *N. otitidiscaviarum *clinical isolates, and other representative species of the *Nocardia *genus**. Sequences of strains isolated in this work are indicated in bold letters. Sequences were aligned using the MUSCLE software version 3.6. The recovered ML phylogram based on the common 1311-nucleotide stretch is shown. Numbers represent ML bootstrap (> 50%). The sequence of *Rhodococcus opacus *was used as out-group. The scale bar indicates 0.01 substitutions per nucleotide position. Accession numbers are given in parentheses. ^T ^means type strain.

To further characterize taxonomically both *Nocardia *isolates, a phylogenetic tree of the 16S rRNA was constructed by using sequences of several clinical *Nocardia *species. Pairwise evolutionary distances were computed using PHYML version 2.4.4 to find the maximum likelihood (ML) tree. The robustness of the inferred tree was assessed with bootstrapping (BP; 1000 pseudoreplicates) also using PHYML  (Figure 2). The *N. otitidiscaviarum *species isolated in this work (accession no. EU203569 and EU203570) are grouped with another clinical isolates of *N. otitidiscaviarum *(accession no. AF430068, AF430067). The other *N. otitidiscaviarum *clinical species, and other nocardiae species belong to different phylogenetic groups (see Figure 2 and below).

## Discussion

Nocardiosis is an acute or chronic infection caused by aerobic, soil inhabiting actinomycetes of the *Nocardia *genus. Patients with nocardiosis are generally debilitated by other underlying diseases such as cancer, pulmonary tuberculosis, hematological malignancies, immunosuppressant therapy, or HIV infection [5,6]. Like the case we report, some patients with pulmonary nocardiosis have COPD and have often been treated with long-term, high-dose corticosteroid therapy. Brain abscess usually arises from hematogenous spread from a primary lung infection. Nocardial brain abscesses are uncommon (< 2%), but are the most common non-pulmonary site in disseminated nocardiosis and have been recorded in between 10 and 15% of patients with this infection [5]. To date and including our case, only six cases of central system nervous infection by *N. otitidiscaviarum *have been reported in the literature [5,7-10], without any taxonomic characterization using molecular techniques such as 16S rRNA gene sequencing.

*N. otitidiscaviarum *was first recognized in samples taken from a Sumatran cavy or guinea pig with ear disease [11]. Infections due to *N. otitidiscaviarum *appear to be quite rare (3%) compared to those caused by other *Nocardia *species and no published reports of human infection appeared until the mid 1960s. The first systemic infection in humans was reported in 1974 [7,12]. This low incidence has been attributed either to reduced pathogenicity or its lower prevalence in soil [5]. It is also possible that *N. otitidiscaviarum *infections are more common, but not properly diagnosed, as postulated by Patel *et al*., [13]. This has also been the case with other *Nocardia *species [14,15].

The diagnosis of nocardiosis, based on conventional phenotypic characterization of the strains (chemotaxonomic, morphology, etc) is very time consuming compared to genotypic methods [16]. Sequence analysis of the 16S rRNA gene has become the "gold standard" for identifying nocardiae isolates at the species level [17]. In our study, PCR amplification of the complete 16S rRNA gene and subsequent RFLP analysis enabled us to obtain band patterns for comparison of the different isolates from the same patient. A 16S rRNA phylogenetic tree has shown that the *N. otitidiscaviarum *species isolated in this work (accession no. EU203569 and EU203570) are grouped with other clinical isolates of *N. otitidiscaviarum *(see above and Figure 2), which correspond to well characterized collection strains (accession no. AF430068 and AF430067). The two isolates obtained from the reported patient exhibited identical 16S rRNA gene sequences to the 16S rRNA gene of *N. otitidiscaviarum *strain DSM 44565 (accession no. AF430068) previously described as genotype II of *N. otitidiscaviarum *[16]. Also, the comparison between 16S rRNA from both isolates and type strain (accession no. AF430067) resulted in a 99.9% identity. Therefore, both isolates can be named as *N. otitidiscaviarum*, although an unambiguous identification to the species level will require additional molecular biological and phenotyping experiments. The other *N. otitidiscaviarum *clinical species reported by other authors belong to different clusters: one comprised of four members (accesion no. Z82237, Z82234, Z82238, Z82239) and another one consisting of two (AB201303, AB201298) (Figure 2). These clusters are separated from the cluster which harbour the *N. ottidiscaviarum *type strain, suggesting that more in deep taxonomic studies of the clinically significant species are necessary [13].

TMP/SMX or minocycline are the most frequently used antibiotics for nocardiosis treatment in localized or mild cases. Combination therapy with a carbapenem or a third-generation cephalosporin with or without amikacin is generally recommended for severely ill patients or for those with central system nervous involvement, in whom mortality is close to 50% when sulfonamide monotherapy is received [18]. Nonetheless, clinical experience with linezolid in nocardiosis is limited [19]. The MIC_90 _for linezolid was ≤ 4 μg/ml against isolates of *N. otitidiscaviarum *[20]. This agent was successfully used to treat individuals with nocardiosis, including patients who had failed therapy with other antimicrobials. These cases have highlighted the excellent penetration of linezolid into cerebrospinal fluid after intravenous administration of the drug every 12 h [19]. The overall mortality rate for nocardial brain abscess is 47.8% for *N. asteroides *infection [21]. In all reported cases of *N. otitidiscaviarum *brain abscess, including our case, the patients died despite surgical and/or antibiotic therapy [7-10] except one case [5] that survived following surgical excision and antibiotic treatment with imipenem and TMP/SMX. Overall mortality in these six cases was 83.3%. In the case we report there are, several factors that may have contributed to the failure to cure the disease, such as deterioration of this patient due to underlying conditions (the patient's age and chronic diseases), as well as the development of severe intolerance to antimicrobials. Aspiration or excision of the abscess would have been necessary to achieve a positive outcome.

In summary, to the best of our knowledge this is the first reported case of *N. otitidiscaviarum *isolated from a patient with pulmonary nocardiosis and brain abscess described and characterized by complete 16S rRNA gene sequencing.

## Consent

We did informed consent for the family's patient about publishing the manuscript to *Annals of Clinical Microbiology and Antimicrobials*. Written informed consent was obtained from the patient for publication of this case report and any accompanying images. A copy of the written consent is available for review by the Editor-in-Chief of this journal.

## Competing interests

We don't have any financial relationship with other people or organizations. And we don't have any financial competing interests that may cause them embarrassment were they to become public after the publication of the manuscript. All authors confirm that this manuscript is original and has not been submitted elsewhere. And all authors confirm that there are any non-financial competing interests to declare in relation to this manuscript.

## Authors' contributions

Each author acknowledges that he has contributed in a substantial way to the work described in the manuscript and its preparation. All authors read and approved the final manuscript.
